# Lipid metabolic reprogramming in cancer cells

**DOI:** 10.1038/oncsis.2015.49

**Published:** 2016-01-25

**Authors:** S Beloribi-Djefaflia, S Vasseur, F Guillaumond

**Affiliations:** 1 INSERM, U1068, Centre de Recherche en Cancérologie de Marseille, Marseille, France; 2 Institut Paoli-Calmettes, Marseille, France; 3 CNRS, UMR7258, Marseille, France; 4 Université Aix-Marseille, Marseille, France

## Abstract

Many human diseases, including metabolic, immune and central nervous system disorders, as well as cancer, are the consequence of an alteration in lipid metabolic enzymes and their pathways. This illustrates the fundamental role played by lipids in maintaining membrane homeostasis and normal function in healthy cells. We reviewed the major lipid dysfunctions occurring during tumor development, as determined using systems biology approaches. In it, we provide detailed *insight* into the essential roles exerted by specific lipids in mediating intracellular oncogenic signaling, endoplasmic reticulum stress and bidirectional crosstalk between cells of the tumor microenvironment and cancer cells. Finally, we summarize the advances in ongoing research aimed at exploiting the dependency of cancer cells on lipids to abolish tumor progression.

## Introduction

**Figure 1:**
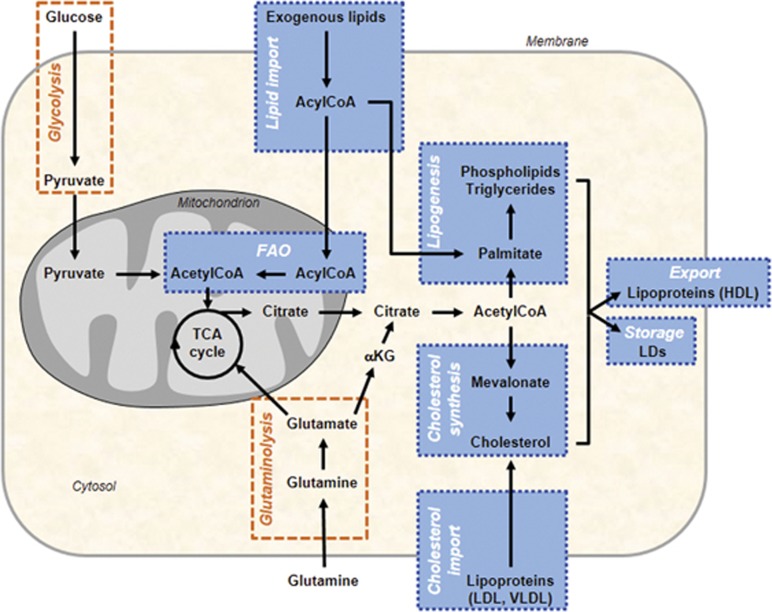
A simplified map of the main altered lipid metabolic pathways in cancer cells. Lipid metabolic network (blue) includes import/export and catabolic pathways (FAO) as well as *de novo* synthesis pathways, such as lipogenesis (that is, synthesis of TGs and PLs) and cholesterol synthesis. Glucose- and/or glutamine-derived citrate, provided by the increased glycolysis and/or glutaminolysis (orange), are common precursors of lipogenesis and cholesterol synthesis. Cancer cells can also take up exogenous cholesterol, transported by LDL and very-low-density lipoproteins (VLDL), to meet their cholesterol requirement. When cholesterol, PLs and TGs are in excess in tumors, they are exported into circulation as high-density lipoproteins (HDLs) or locally stored into LDs. Exogenous FAs taken up by cancer cells are broken down to produce energy through mitochondrial FAO process. TCA cycle, tricarboxylic acid cycle αKG, α-Ketoglutarate.

Metabolic reprogramming is now firmly established as a hallmark of cancer.^1^ Tumors share a common phenotype of uncontrolled cell proliferation and for this they must efficiently generate energy and biomass components in order to expand and disseminate. The required changes in metabolic phenotype are directly driven by successive oncogenic events (oncogene activation and loss of tumor suppressors), and by the constraints imposed by the tumor microenvironment (TME) (poor oxygenation and nutrient scarcity).^2, 3^ Hence, cancer cells show an expanded metabolic repertoire that affords the flexibility to withstand and grow in this harsh tumor environment. The first adaptive events in tumor metabolism to be identified are an exacerbated glucose uptake and glycolysis utilization leading to increased lactate production (that is, the Warburg effect^4^).^5, 6^ Cancer cells also rely on glutamine consumption, which provides carbon and amino-nitrogen needed for amino-acid, nucleotide and lipid biosynthesis.^6, 7^ Functionally dependent on glucose and glutamine catabolic pathways but commonly disregarded in the past, alterations in lipid- and cholesterol-associated pathways encountered in tumors are now well recognized and more frequently described (Figure 1).^8, 9, 10^ Highly proliferative cancer cells show a strong lipid and cholesterol avidity, which they satisfy by either increasing the uptake of exogenous (or dietary) lipids and lipoproteins or overactivating their endogenous synthesis (that is, lipogenesis and cholesterol synthesis, respectively) (Figure 1). Excessive lipids and cholesterol in cancer cells are stored in lipid droplets (LDs), and high LDs and stored-cholesteryl ester content in tumors^11, 12, 13, 14^ are now considered as hallmarks of cancer aggressiveness.^13, 15, 16, 17^ Colon cancer stem cells showed higher LD amount than their differentiated counterparts, as revealed by Raman spectroscopy imaging.^18^ Moreover, LD-rich cancer cells are more resistant to chemotherapy.^11^ Therefore, using Raman-based imaging to define tumor LD content is an emerging tool for monitoring or predicting drug treatment response in cancer patients.^19, 20^ Moreover, LD content, especially cholesteryl ester, is mobilized by pancreatic cancer cells under a restricted cholesterol-rich low-density lipoprotein (LDL) supply^14^ and limiting LDL uptake reduces the oncogenic properties of pancreatic cancer cells and rendered them more sensitive to cytotoxic drugs.^14^ Survival and metastatic spreading of cancer cells also rely on exogenous fatty acid (FA) uptake and consumption, the latter through fatty acid β-oxidation (FAO) pathway, even in cells exhibiting high lipogenic activities (Figure 1).^21, 22, 23^ FAO is considered as the dominant bioenergetic pathway in non-glycolytic tumors, such as prostate adenocarcinoma and diffuse large B-cell lymphoma.^24, 25, 26^ The dependence of cancer cells on FAO is further heightened in nutrient- and oxygen-depleted environmental conditions.^22^ Then, therapeutic strategies designed to exploit the lipid-related metabolic dependence in cancer must be carefully targeted to achieve the desired effect and avoid harmful consequences for normal metabolic functions.

Lipids encompass a vast class of biomolecules of unique chemical structure in terms of FA chain length, number and location of double bonds as well as backbone structures (glycerol and sphingoid bases). The functional consequence of this lipid diversity is still not fully understood. However, lipids have been described to exert multiple biochemical functions during cancer development. Historically, they were viewed as passive components of cell membranes where they form lipid rafts that facilitate signaling protein recruitment and thus protein–protein interactions promoting signal transduction. Important changes in lipid composition (saturated (SFA) vs unsaturated FA) and abundance severely alter membrane fluidity and protein dynamics. For example, an increase in saturated phospholipids (PLs) markedly alters signal transduction, protects cancer cells from oxidative damage such as lipid peroxidation and potentially inhibits the uptake of chemotherapeutic drugs.^27, 28^ In addition to their structural roles, lipids orchestrate signal transduction cascades and can also be broken down into bioactive lipid mediators, which regulate a variety of carcinogenic processes, including cell growth, cell migration and metastasis formation.^29, 30, 31^

In this review, we summarize the major lipid dysfunctions identified in various tumors using gene candidate or ‘-omics' approaches. We focus on the impact of lipid content alterations on intracellular oncogenic signaling and on endoplasmic reticulum (ER) homeostasis. We also detail the lipid exchange between stroma cellular components and cancer cells. Finally, we present advances in the therapeutic targeting of metabolic actors associated with lipid pathways in preclinical and clinical development.

## Lipid reprogramming in tumors

### Lipid alterations identified from tumor-specific gene expression profiling

Candidate-gene expression studies identified upregulated transcripts involved in lipogenesis and cholesterol synthesis pathways (Figure 1), which are essential for development and progression of a wide variety of tumors. Increased expression of lipogenic enzymes, such as acetyl-CoA carboxylase (ACC) and fatty acid synthase (FASN), and ATP citrate lyase (ACLY) that promote also cholesterol synthesis, represent a nearly-universal phenotypic alteration in most tumors.^32, 33^ FASN overexpression predicts poor prognosis in cancer patients.^34^ Its expression levels appear at the precancerous lesion stage and persist in metastatic breast and prostate tumors.^34^ As these initial observations, many other candidate genes, involved in cholesterol-related pathways (uptake, synthesis and storage) and FAO, proved to be crucial in supporting malignancy.^8, 10, 35^ FAO-limiting enzymes, the carnitine palmitoyltransferase 1 isoforms A and C (CPT1A and C) are overexpressed in many human tumors.^36, 37, 38^ CPT1C upregulation, induced by AMPK and p53, has been shown to protect cancer cells from death when they are under deprived glucose and oxygen conditions.^36, 38, 39^ Inversely, knockdown of CPT1 sensitizes cancer cells to radiotherapy and apoptosis inducers.^40, 41, 42^

Our large-scale microarray profiling, centered on metabolic genes, reveals lipid pathways as the most altered metabolic routes in pancreatic tumors, especially activated cholesterol and LDL metabolisms.^14^ These tumors harbor also specific alterations in metabolic pathways related to lipid messengers (phosphatidylinositols, PIs), lipid mediators (leukotrienes) and structural lipids (glycosphingolipids).^14^ This lipid signature unravels the high dependence of pancreatic tumors on cholesterol and identifies exogenous cholesterol uptake, through LDLR, as the major cholesterol pathway mediating tumor growth. Colorectal cancer (CRC) lipid signature, defined from a limited lipid-related genes expression profiling, reveals four genes (ABCA1, ACSL1, AGPAT1 and stearoyl-CoA desaturase (SCD)) overexpressed only in stage II CRC patients with a high risk of relapse. This signature displays stronger power and accuracy than the currently used clinical classification.^43^

### Lipid alterations identified from tumor-specific lipid profiling

Recent advances in lipid analytical and imaging technologies, including electrospray ionization, matrix-assisted laser desorption/ionization, tandem mass spectrometry (MS/MS) and Raman scattering microscopy, have greatly progressed such lipidomic analysis.^44^ Raman-based imaging offers lipid compositional mapping of cellular compartments, such as LDs.^26, 45^ These complementary approaches provide crucial information on tumor lipid phenotype, in particular abundance, FA composition and spatial distribution of lipid classes within tumors. Over the past few years, much effort has surrounded establishing PL signature of malignant tumors. This signature segregates malignant tumors from their benign counterparts as well as localized tumors from advanced ones. Indeed, breast tumors, when compared with adjacent normal tissue, have been characterized by a striking increase in membrane phosphatidylcholine and phosphatidylethanolamine and in PL-induced cell signaling, PI.^46, 47^ In addition to these changes in PL amounts, the phosphatidylcholine content was found to be enriched in SFA, and this phosphatidylcholine composition was correlated with high tumor grade and poorer overall survival.^46^ This membrane lipid saturation, a feature shared by all lipogenic tumors,^27^ reduced membrane fluidity and dynamics^48^ and increased chemotherapy resistance.^27^ The specific PI signature revealed a shift toward polyunsaturated FA chain composition in PI from invasive breast cancers when compared with that in PI from *in situ* carcinoma.^49^ These findings highlight significant differences in FA composition depending on PL class and tumor grade. Unlike breast tumors, the lipid signature of *Myc*-induced lymphoma is characterized by reduced phosphatidylserine, phosphatidylethanolamine and PI amounts and by elevated monounsaturated FA-phosphatidylglycerol (PG) levels when compared with normal tissues.^50^ The increased PG is also found in renal cell and hepatocellular carcinomas.^51, 52^ PG serves as a precursor of cardiolipin, which is found almost exclusively in mitochondrial membranes and intimately involved in maintaining mitochondrial functionality and membrane integrity. An abnormal cardiolipin molecular species distribution and a decrease in CL content in brain tumor mitochondria, revealed by shotgun lipidomic analysis, lead to irreversible respiratory injury and may impede the use of alternative energy sources to glucose.^53^

Lipidomic profiling has revealed unsuspected and recurrent lipid changes at the class and molecular species levels in cancer cells. As previously discussed, PL-specific composition may help to discriminate low- and high-grade tumors as well as malignant cells from benign ones.^46, 47, 49, 50^ Moreover, combined with transcriptome/proteome analyses, lipidomic data could also unravel new potential lipid-related targets for drug development or new treatments combining inhibitors of these targets with currently used chemotherapy.

## Lipid rafts in cancer cell signaling

### Increased lipid rafts in tumors

**Figure 2:**
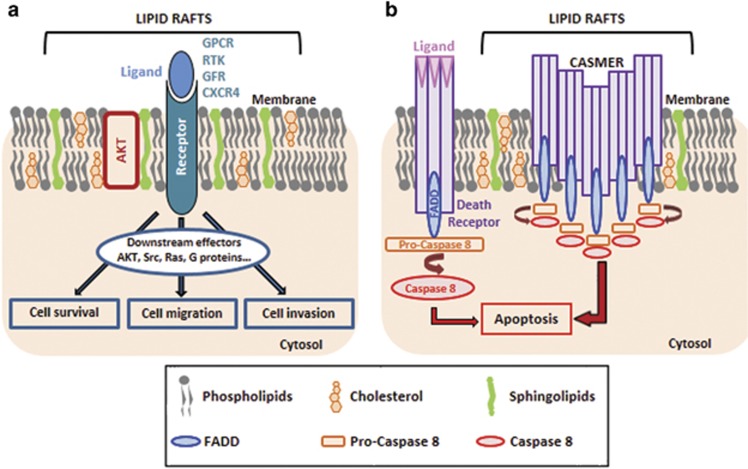
Lipid rafts as platforms for cell signaling. (**a**) Lipid rafts are formed by a phospholipid bilayer enriched in cholesterol, sphingolipids and resident signaling proteins (AKT) and receptors (GPCR, G protein-coupled receptor; RTK, receptor tyrosine kinase including growth factor receptor (GFR); CXCR4, C-X chemokine receptor 4). Once activated by their respective ligands, the receptors recruit different signaling effectors that promote cell survival, cell migration and cell invasion, all of which contribute toward tumor growth. (**b**) Aggregation of death receptors (DR4/DR5, Fas) in lipid rafts forms CASMERs. Recruitment of CASMERs in a restricted space enhances fas-associated protein with death domain (FADD)/Caspase-8 death signaling pathway when compared with apoptotic signal induced by the activation of non-clustered death receptors.

Cell membranes contain different classes of lipids, some of which, in particular cholesterol and sphingolipids, form specific planar microdomains known as lipid rafts (Figure 2).^54^ These differ from the cavin and caveolin protein-enriched invaginated-lipid rafts known as caveolae.^28^ Both are essential not only for membrane protein dynamics and trafficking but also for cell survival and cell death program execution.^55^ In cancer cells, a wide range of signaling proteins and receptors regulating pro-oncogenic and apoptotic pathways during the early, advanced and metastatic stages of carcinogenesis reside in lipid rafts (Figure 2).^55^ Moreover, lipid rafts/caveolae and their main component, cholesterol, are enhanced in membrane of multiple cancer cells^56, 57, 58, 59, 60^ as well as in membranes of tumor-released exosomes.^61^

### Impact of disrupted lipid raft integrity on tumor cell fate

Decreasing cholesterol content with membrane-depleting agents (methyl-β-cyclodextrin) or cholesterol synthesis inhibitors (statins) helped to decrypt the oncogenic signaling pathways whose activation is entirely dependent on lipid raft integrity. Anchored-lipid raft AKT protein has been extensively investigated in cancer cells (Figure 2a).^62, 63^ Its aberrant activation, contributing to tumor development and invasiveness,^64, 65^ correlates with increased lipid rafts in cancer cells.^66, 67^ Lipid raft disruption inhibits AKT activation^63, 67, 68^ and then reduces tumor cell proliferation (Figure 2a).^66^ Lipid rafts also exert crucial roles in cancer dissemination. By regulating cytoskeletal reorganization and focal adhesion dynamics, lipid rafts regulate cancer cell migration.^69, 70^ They are also important for ligand-directed migration of T-lymphoblastic lymphoma cells by maintaining C-X-C chemokine receptor type 4 (CXCR4) dimer conformation.^71^

Novel raft-based entities, known as clusters of apoptotic signaling molecule-enriched rafts (CASMERs), have been recently described (Figure 2b).^55^ These are constituted by co-aggregation of lipid rafts with death receptors (Fas/CD95, tumor necrosis factor-related apoptosis-inducing ligand or TRAIL) and their downstream apoptotic molecules. This configuration activated efficiently the apoptotic response independently of death receptor ligands (FasL and TNFα) (Figure 2b). CASMER formation and its subsequent Fas/CD95 or TRAIL-induced cell death can be inhibited by cholesterol-depleting agents, as described in leukemia cells and non-small cell lung carcinoma.^55, 72^ Similarly, resveratrol induced-CASMER formation and sensitization of colon carcinoma cells to death receptor-mediated apoptosis are prevented by cholesterol membrane depletion.^73^

These findings provide evidence for multiple oncogenic events depending on lipid raft integrity. Disruption of these microdomains, which act as hubs linking receptors to their signaling effectors, thus represents a valid therapeutic strategy in cancer treatment.

## Complex lipid and cholesterol alterations inducing ER stress

### Cancer cell fate following persistent ER stress

The ER ensures protein folding and maturation as well as calcium homeostasis and regulates lipid metabolic processes. Accumulation of misfolded proteins, membrane lipid saturation or imbalanced calcium homeostasis leads to ER stress (ERS) and activation of the unfolded protein response (UPR).^74^ UPR is transduced through three distinct ERS sensor proteins: ATF6 (activating transcription factor 6), PERK (protein kinase RNA-like endoplasmic reticulum kinase) and IRE1 (inositol-requiring transmembrane kinase/endonuclease 1), which either reduce protein translation or increase ER-associated protein degradation to maintain cell survival. UPR can evoke cell-cycle arrest in G1 phase leading to the accumulation of quiescent cancer cells awaiting a more permissive environment to re-enter the cell cycle.^75^ When cancer cells are submitted to persistent stresses (that is, hypoxia, membrane lipid saturation and nutrient deprivation), UPR leads to cell death.

### Changes in complex lipid/cholesterol content and composition cause ERS-induced apoptosis

Membrane PL saturation disturbs ER structure^66^ and then impairs ER homeostasis,^76, 77^ a phenomenon commonly encountered in various cancer cells.^27^ Lipid saturation, induced by the loss of the enzyme SCD1, was shown to promote ERS-activated apoptosis.^78^ A similar cancer cell fate is noticed following inactivation of sterol regulatory element-binding protein, the major transcriptional regulator of lipogenic genes, in a lipid-poor environment.^79^ This lipotoxic effect is abrogated by addition of exogenous unsaturated lipids^80^ or by re-expressing SCD1.^79^ Recently, imbalanced cholesterol homeostasis, leading to free cholesterol (FC) overload, was shown to induce ERS in cancer cells. Indeed, FC accumulation in HepG2 cells, induced by antitumor alkylphospholipids (perifosine, miltefosine and edelfosine),^81^ triggers an increase in the ERS marker, CHOP (C/EBP homologous protein).^82^ Similarly, inhibitors of cholesterol esterification, targeting the enzyme sterol-O-acyl transferase 1 (SOAT1), activated ERS markers in adrenocortical adenocarcinoma cells.^83^ High ceramide levels in ER, resulting from an increase either in membrane sphingomyelin hydrolysis or in ceramide *de novo* synthesis, can also induce ERS.^84, 85^ Cannabinoids, by increasing synthesized ceramide content, trigger ERS-induced cell death in human glioma and pancreatic adenocarcinoma.^86, 87, 88^ This process results from a p8-dependent upregulation of CHOP/ATF4 branch of UPR.^87^ Finally, increased exogenous ceramide uptake leads to apoptosis in various human cancer cells, including head and neck squamous carcinoma cells^89, 90^ and salivary adenoid cystic carcinoma cells.^91^

Data demonstrating ERS-induced apoptosis in cancer cells submitted to complex lipid and/or cholesterol homeostasis alterations open a promising therapeutic window. It allows us to predict that manipulating cholesterol and lipid supplies or metabolic pathways leading to PL saturation, FC or ceramide accumulation may impede tumor growth and dissemination.

## Tumor-stroma communication mediated by lipids

In human cancers, the TME, formed by extracellular matrix components and numerous stromal cells including cancer-associated fibroblasts (CAFs), infiltrating immune cells, adipocytes, nerve cells, vascular/lymphatic endothelial cells, represents up to 90% of the tumor mass.^92^ A molecular dialog between cancer cells and adjacent CAFs or immune cells has been clearly demonstrated to support tumor growth and progression. Today, the central role played by bioactive lipids and FAs as mediators of this crosstalk between cancer cells and stroma is increasingly recognized.

### Cancer-stroma interplay through free FAs

**Figure 3:**
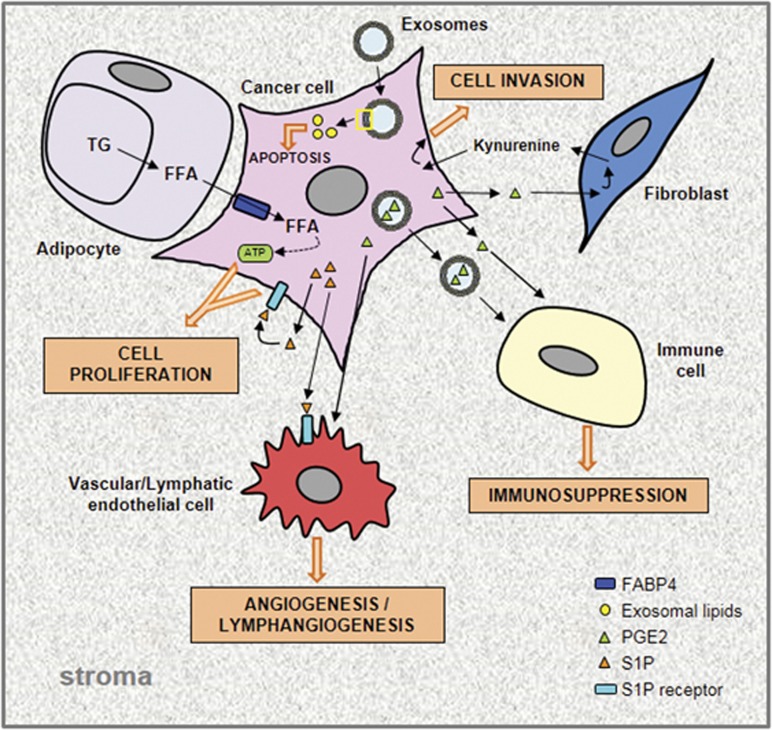
Tumor–stroma bidirectional dialog. Schematic representation of lipid exchanges between cancer cells and the different cell types found in the TME. In adipocytes adjacent to cancer cells, the hydrolysis of TG, stored in LDs, releases free fatty acids (FFAs) which are taken up by cancer cells, transported through fatty acid binding protein 4 (FABP4) and degraded to provide ATP needed for their growth. Bioactive lipids secreted by cancer cells, PGE2 and S1P, exert their effects on stromal cells through paracrine mechanisms. The PGE2, transported or not by exosomes, promotes angiogenesis and also immunosuppression. The latter effect results from an activation of myeloid-derived suppressor cells and differentiation of monocytes into suppressor macrophages. Moreover, tumor-derived PGE2 induces kynurenine secretion by CAFs which in turn promote cancer cell invasiveness. S1P, by its binding on its specific receptor, promotes cancer cell proliferation and angiogenesis/lymphangiogenesis in an autocrine and paracrine manner, respectively. Taken together, FFA and free bioactive lipids contribute toward promoting tumor growth. Exosomes in TME contain high lipid levels within the membrane and lumen, and therefore constitute extracellular lipid sources which can be internalized by cancer cells and are responsible for the increased cell lipid concentration which triggers an ERS-induced cell death.

Numerous tumors grow in the vicinity of adipocytes or metastasize to adipocyte-rich host environment. Metastatic ovarian cancer cells home to omental adipose tissue, which constitutes an important reservoir of triglycerides (TGs).^93^ Hydrolysis of these TG provides free FA (FFA), which are taken up and used as energy source by metastatic ovarian cancer cells (Figure 3).^93^ A similar FA exchanges also exist between adipocytes and metastatic bone marrow-derived prostate cancer cells.^94^ This adipocyte-cancer cell dialog is an adaptive metabolic process set up by cancer cells to take full advantage of the lipids stored in TME cells. FA translocation from stromal cells to cancer cells can be mediated by lipoproteins, serum albumin and exosomes. It is tempting to speculate that FA carried by serum albumin could be taken up by cancer cells through macropinocytosis: a non-receptor mediated endocytosis process constituting part of an ancestral strategy used to salvage extracellular nutrients.^95, 96^ Exosomes can also serve as carriers of FA and are taken up by recipient cells (Figure 3). Their content, similar to that of parental cells, is mostly enriched in SFA more than monounsaturated FA and polyunsaturated FA, the latter group of which is most represented by arachidonic acid, the precursor of eicosanoids (prostaglandins and leukotrienes).^97^ Once internalized, exosomes transfer their lipid material to the receiving cell.^98^ The ensuing lipid accumulation alters lipid homeostasis thereby triggering ERS-induced apoptosis and/or disturbing lipid raft signaling, as discussed in the previous section. Beloribi *et al.*^99^ also demonstrated that synthetic exosome-like nanoparticles, mimicking the lipid composition of cancer exosomes, inhibit the Notch survival pathway leading to differentiated pancreatic cancer cell death (Figure 3).

### Tumor–stroma dialog orchestrated by prostaglandins

An increase in prostaglandins (PGs) in cancer cells not only promotes tumor growth in a paracrine manner, but also coordinates the complex dialog between tumor cells and the surrounding stromal cells. This crosstalk evades the immune system attack by promoting immunosuppression (Figure 3).^30^ Breast tumor-derived prostaglandin E2 (PGE2) has been shown to induce, through an exosome-dependent transport, myeloid-derived suppressor cell activation, which in turn promotes tumor growth.^100, 101^ Moreover, PGE2 was found to promote the differentiation of monocytes into tumor-associated suppressive macrophages in cervical tumors.^102^ A pro-angiogenic activity of tumor-derived PGE2 has also been demonstrated in different cancers.^30, 103, 104, 105^ PGs released from cancer cells expressing the rate-limiting enzyme of PG synthesis, cyclooxygenase-2 (COX-2), trigger endothelial cell migration *in vitro* and neovascularization *in vivo.*^103, 104^ Recently, a PGE2-dependent dialog between breast tumor cells and CAFs has been demonstrated. Tumor-derived PGE2 activates the CAF-dependent secretion of a tryptophan catabolite, the kynurenine, which in turn increases cancer cell invasiveness (Figure 3).^106^

### Sphingolipid derivative as a mediator of tumor–stromal cell communication

Sphingosine-1-phosphate (S1P), another bioactive lipid secreted by cancer cells, induces angiogenesis and lymphangiogenesis through its binding on S1P receptor 1, and facilitates tumor growth and metastasis formation.^107, 108^ Moreover, high extracellular S1P levels, induced by overexpression of the upstream regulatory sphingosine kinase, increases migration and tube formation in co-cultured vascular or lymphatic endothelial cells (Figure 3).^109^

Together, these findings highlight the crucial role of lipids and their modes of transport in supporting the tumor-TME dialog, which is essential for tumor cell proliferation and dissemination.

## Lipids as cancer therapy targets

### Targeting the lipid and cholesterol dependence of cancer cells

Inhibitor agents directed against lipogenic enzymes (FASN, ACLY and ACC) have been the subject of numerous studies; and their efficacy as anticancer therapies have been proven in various preclinical models of carcinogenesis (Table 1).^110, 111, 112^ However, high adverse side effects of FASN-targeting drugs have precluded their clinical development. Numerous studies, using pharmacological agents targeting liver X receptor (LXR), a crucial transcriptional regulator of cholesterol homeostasis, have shown relevant anticancer roles but also with undesired side effects.^113^ Recently, an LXR inverse agonist (SR9243), devoid of toxic side effects and with similar impacts on colon cancer, holds significant promise for cancer therapy (Table 1).^114^ Alternative therapies directed against SCD1 enzyme have shown a delay in tumor growth in various mouse xenograft models.^115^ Interestingly, the high dependency of cancer cells on SFA can be exploited to increase tumor-drug delivery, as loading drugs in liposomes enriched in saturated phosphatidylcholine has been shown to reduce the metastatic spread of pancreatic cancer *in vivo.*^116^ The use of CPT1 inhibitors (that is, etomoxir or ranolazine) provides beneficial effects in FAO-dependent tumors, notably in prostate cancer^42^ and in human leukemia when they are combined with pro-apoptotic agents.^40^ Recently, a novel CPT1a inhibitor, ST1326, has been shown to drive leukemia cells toward apoptosis. This apoptotic effect results from an accumulation of palmitate.^37^ Several strategies have been developed to target cholesterol or cholesterol/isoprenoid synthesis. Oxidosqualene cyclase inhibitor (Ro 48-8071)^117^ or statins reduced tumor growth, angiogenesis and metastasis incidence in mouse carcinogenesis models (Table 1).^118^ However, despite promising preclinical results, the use of statins as monotherapy failed to improve patient outcome in many cancers^119^ because in addition to inhibiting cholesterol synthesis, statins increase circulating cholesterol supply through LDLR. In contrast, cholesterol depletion in high LDLR-expressing cancer cells by combining chemotherapy with the blockade of LDLR represents a promising alternative therapeutic option to limit pancreatic tumor growth. Indeed, LDLR silencing potentiates tumor regression induced by chemotherapy.^14^ Finally, pharmacological inhibitors of SOAT1 enzyme (avasimibe, Sandoz 58-035) (Table 1), through limiting cholesteryl ester storage, have been shown to suppress tumor growth in prostate cancer xenograft models.^13^

### Lipid raft targeting

As discussed above, anticancer drugs that disturb membrane cholesterol content can be used to impair lipid raft-dependent cell survival or cell death pathways. Methyl-β-cyclodextrin depletes membrane cholesterol and inhibits human melanoma, breast and ovarian cancer growth without elicited acute systemic cytotoxicity (Table 1).^68, 120^ Moreover, when combined with tamoxifen, methyl-β-cyclodextrin slows down melanoma cancer progression by inhibiting AKT and favoring drug uptake,^121^ probably through increased membrane permeability. By increasing cholesterol ABCG1-dependent efflux, LXR agonist abrogates the lipid raft-dependent AKT survival pathway and then induces prostate cancer cell apoptosis (Table 1).^122^ In glioblastoma, such LXR agonist induces tumor cell death *in vivo*; an effect resulting from an increase in both LDLR degradation and ABCA1-dependent cholesterol efflux.^123^ Other pharmacological treatments known to reduce lipid raft-associated cholesterol, such as inhibitors of cholesterol synthesis, have been shown to promote prostate cancer growth arrest and cell death.^67, 124^ Finally, different therapeutic drugs, promoting CASMER formation, are being investigated. By their accumulation in cell membrane, synthetic alkylphospholipids (edelfosine, perifosine)^125, 126^ and plant-derived compounds (Avicin D, resveratrol)^127, 128^ promote the recruitment of death receptors, including Fas and TRAIL, into lipid rafts (Table 1). This results in the activation of ligand-independent Fas and/or TRAIL apoptotic pathways in various cancer cells (Figure 2).

### Therapies promoting ERS-induced apoptosis

The disruption of lipid homeostasis induces ERS and then cancer cell death when the ERS exceeds the cell's adaptive mechanisms. Nelfinavir and its analogs inhibit Site-1 and Site-2 proteases (S1P and S2P), both of which are required for the release of the mature and transcriptionally-active form of sterol regulatory element-binding protein-1. The ensuing decrease in lipogenic gene expression induces ERS and apoptosis in liposarcoma^129^ and castration-resistant prostate cancer cells.^130^ This compound associated with chemotherapy is currently in phase II clinical trials for myeloma, glioblastoma, pancreatic and lung cancer (Table 1).^131^ Other chemical compounds, Orlistat and C75, by abrogating the activity of the rate-limiting enzyme of lipogenesis FASN, trigger activation of the UPR and cell death in prostate cancer cells (Table 1).^132^ Recently, mitotane has been demonstrated to have an anticancer role, impeding cholesterol esterification by inhibiting SOAT1 enzyme and consequently inducing an overload of cytotoxic FC within adrenocortical carcinoma cancer cells.^83^ This alteration in lipid homeostasis causes ERS-induced apoptosis and seems to be specific to steroidogenic cancer cells. A similar effect, with lower efficacy, is observed with the Sandoz 58-035 SOAT inhibitor (Table 1).^83^ As prostate tumors also exhibit high SOAT1 expression levels, it is possible that mitotane may promote ERS-induced apoptosis. Preclinical investigations are ongoing on the use of an inhibitor of SCD1 (A939572) which triggers SFA accumulation, in clear cell renal cell carcinoma. Its combined administration with tyrosine kinase or mTOR inhibitors appears to improve its efficiency and reduce its cytotoxicity.^133^ Impaired HIF2α/PLIN2-dependent lipid storage in clear cell renal cell carcinoma disturbs ER homeostasis and enhances sensitivity to ERS-inducing agents.^11^ Hence, coupling proteasome inhibitors, such as bortezomib known to induce the UPR,^134^ with HIF2α-specific inhibitors currently under development for treating clear cell renal cell carcinoma patients, could be a rational therapeutic approach. Finally, treatments leading to ceramide accumulation and then ERS-induced apoptosis have shown encouraging results in many preclinical cancer models (Table 1).^135, 136^ Indeed, cannabinoid receptor agonists, supporting ceramide-dependent pro-apoptotic cascade, in combination with conventional chemotherapy could be therapeutically exploited for the management of glioblastoma.^136^

### Disrupting the lipid-mediated dialog between cancer cells and TME cells

Targeting either the lipid messengers or their carriers between stromal and tumor cells constitutes an interesting anticancer therapeutic route to continue investigating. The use of COX-2 enzyme inhibitor, Celecoxib, to disrupt PG synthesis has revealed its strong antitumoral and antimetastatic effect in various preclinical models.^137, 138^ Moreover, it can attenuate patient chemoresistance as well as undesired side effects of anticancer drugs in various cancers.^137, 139, 140, 141, 142^ One clinical study on breast cancer patients is ongoing to evaluate the effect of Celebrex (that is, celecoxib) alone or in combination with vitamin D (Clinical Trial No NCT01769625). New COX-2 inhibitors with lower adverse side effects, such as CG100649,^143^ and antagonists of PGE2 receptors^30^ have shown promising results in multiple cancer preclinical models. Regarding the bioactive lipid S1P, its neutralizing monoclonal antibody, sphingomab, has proven to be effective at inhibiting angiogenesis, tumor growth and metastasis in multiple cancer cell lines.^107^ Similar effects on cancer progression have been observed with inhibitors of its upstream regulatory sphingosine kinase 1 (SphK1)^144^ or its receptor (S1P receptor 1).^145^ Moreover, sphingomab increases the sensitivity of RCC-bearing mice to sunitinib treatment, which inhibits VEGFR2 tyrosine kinase.^146^ Hence, sphingomab constitutes a promising therapy for RCC non-responder patients. In the extracellular space, all these bioactive lipids can be found within exosomes, hence alternative strategies aiming at decreasing exosome generation and secretion or modifying the exosome content within tumors, should be considered in the future as potential cancer treatments.

## Conclusion

Compelling evidence gained from untargeted/targeted lipidomics studies, cancer preclinical models and clinical trials, has revealed the crucial role of lipid classes and molecular species in supporting tumor growth and metastatic dissemination. Disrupting lipid metabolic pathways to unbalance lipid homeostasis, through the targeting of enzymes, receptors or bioactive lipids, induces tumor regression and inhibits metastatic spread. These effects result from: (1) fundamental changes in lipid raft composition; or (2) sustained ERS-induced UPR, both leading to cancer cell death; or (3) disruption of the lipid-mediated crosstalk between stromal and tumor cells, impeding the pro-tumoral function of stromal cells. Continued efforts to identify all the key actors within these different processes may offer novel metabolic targets for cancer treatment. These clinical strategies, based on the tumor dependence towards lipids, may hold promise for cure the most intractable cancers, including pancreatic and lung cancers, which will become the two deadliest cancers in horizon 2030.

## Figures and Tables

**Table 1:** Non-exhaustive list of lipid-related drugs under study for cancer treatment

*Target*	*Drug*	*Drug development stage*	*Cancer type*	*References*
*Targeting lipid and cholesterol dependencies in cancer cells*				
FASN	Cerulenin	Preclinical	Solid tumors	Reviewed in Flavin *et al.*^110^
	Orlistat	Preclinical		
	C75	Preclinical		
	Triclosan	Preclinical		
	EGCG	Preclinical		
ACLY	SB-204990	Preclinical	Solid and non-solid tumors	111
ACC	TOFA	Preclinical	Ovarian cancer	112
LXR	T0901317	Preclinical	Solid tumors	113
	SR9243	Preclinical	Colon cancer	114
SCD1	A939572	Preclinical	Solid tumors	115
	CAY-10566	Preclinical	Solid tumors	
CPT1	Etomoxir, Ranolazine	Preclinical	Prostate cancer	42
			Leukemia	40
	ST1326	Preclinical	Leukemia	37
OSC	Ro 48-8071	Preclinical	Pancreatic and colon cancers	117
HMGCR	Statins	Preclinical and clinical	Solid tumors	Reviewed in Clendening and Penn^118^
SOAT1	Avasimibe Sandoz 58-035	Preclinical	Prostate cancer	13

*Modulating lipid raft components to induce cell death signaling*				
Cholesterol	Methyl-β-cyclodextrine	Preclinical	Melanoma, breast and ovarian cancers	68, 120, 121
LXR	T0901317	Preclinical	Prostate cancer	122
	GW3965	Preclinical	Glioblastoma	123
HMGCR	Simvastatin	Preclinical	Prostate cancer	67
Cell membrane	Perifosine	Phase I–III	Solid and non-solid tumors	Reviewed in Pachioni Jde *et al.*^147^
	Edelfosine/Perifosine	Preclinical	Leukemia, lymphoma, mantel lymphoma	126
Death receptors (Fas/TRAIL)	Avicin D	Preclinical	Solid tumors	Reviewed in Wang *et al.*^148^
	Resveratrol	Preclinical	Solid tumors	Reviewed in Tomé-Carneiro *et al.*^149^
		Phase I–III	Colon, colorectal and hepatic cancers	

*Disrupting lipid homeostasis to induce ER stress and apoptosis*				
Site-1 and Site-2 proteases	Nelfinavir	Phase II	Myeloma, glioblastoma, pancreatic and lung cancers	131
FASN	Orlistat C75	Preclinical	Prostate cancer	132
SOAT1	Mitotane Sandoz 58-035	Preclinical	Adrenocortical carcinoma	83
SCD1	A939572	Preclinical	Clear cell renal cell carcinoma	133
Ceramide accumulation	Cannabinoids	Preclinical	Solid tumors	135
			Glioblastoma	136

*Targeting lipid mediators of tumor–stroma dialog*				
COX-2	Celecoxib	Preclinical Ongoing clinical trial (n°NCT01769625)	Solid tumors Breast cancer	137, 139, 140, 141, 142
	CG100649	Preclinical	Colorectal cancer	143
PGE2 receptors	SC-51322	Preclinical	Esophageal adenocarcinoma,	Reviewed in Wang and Dubois^137^
	AH6809	Preclinical	colorectal and lung cancers	
	AH23848B	Preclinical		
	ONO-AE3-208	Preclinical		
	ONO-8711	Preclinical		
S1P	Sphingomab	Preclinical	Solid and non-solid tumors	107
			Renal cell carcinoma	146
SphK1	SK1-I	Preclinical	Breast cancer	144
S1PR1	FTY720	Preclinical	Colorectal cancer	145

Abbreviations: ACC, acetyl-CoA carboxylase; ACLY, ATP citrate lyase; COX-2, cyclooxygenase-2; CPT1, carnitine palmitoyltransferase 1; FASN, fatty acid synthase; LXR, liver X receptor; HMGCR, 3-hydroxy-3-methylglutaryl CoA reductase; OSC, 2,3-oxydosqualene lanosterol cyclase; PGE2, prostaglandin E2; SCD1, stearoyl-CoA desaturase-1; SOAT1, sterol-O-acyl transferase 1; SphK1, sphingosine kinase 1; S1PR1, S1P receptor 1; TRAIL, tumor necrosis factor-related apoptosis-inducing ligand.
